# Fiber-Reinforced Coral Aggregate Concrete: A Review of Mechanical, Dynamic, and Durability Properties

**DOI:** 10.3390/ma19040765

**Published:** 2026-02-15

**Authors:** Yuliu Wei, Mohd Nizam Shakimon, Peihuan Ye, Yuliang Chen

**Affiliations:** 1 Faculty of Engineering, Science & Technology, Kuala Lumpur University of Science and Technology, Kajang 43000, Malaysia; yuliu.wei@gxust.edu.cn; 2 College of Civil and Architecture Engineering, Guangxi University of Science and Technology, Liuzhou 545006, China; peihuan.ye@gxust.edu.cn (P.Y.); ylchen@gxust.edu.cn (Y.C.); 3 Guangxi Zhuang Autonomous Region Engineering Research Center of Assembly Structure Safety Prevention and Control, Liuzhou 545006, China; 4 Liuzhou Key Laboratory of Green Advanced Civil Engineering Materials Application, Guangxi University of Science and Technology, Liuzhou 545006, China

**Keywords:** coral aggregate concrete, fiber reinforcement, mechanical properties, complex loading conditions, durability

## Abstract

Coral aggregate concrete (CAC) is a promising sustainable material for construction on remote islands, but it is often limited by relatively low strength and durability. Fiber reinforcement has therefore been introduced as an effective modification strategy. This review focuses on fiber-reinforced coral aggregate concrete (FRCAC), highlighting the roles of different synthetic and natural fibers in improving its performance. Firstly, the characteristics of coral aggregates and the effects of seawater mixing are summarized. Then, the influence of fiber incorporation on the mechanical behavior of CAC under static loading, including compressive, tensile, and flexural responses, is reviewed. In addition, the performance of FRCAC under dynamic and complex loading conditions, such as impact, cyclic, and triaxial loading, is discussed. Overall, fiber reinforcement significantly enhances the tensile strength, ductility, and energy dissipation capacity of CAC, particularly at high strain rates. The maximum reported improvements in splitting tensile strength and flexural strength can reach up to approximately 58% and 68%, respectively, depending on fiber type and dosage. However, the enhancements in compressive strength and elastic modulus are generally limited, with maximum reported increases of about 23% and 7%, respectively. Under multiaxial stress states, fibers mainly contribute to crack control and damage mitigation rather than substantial strength enhancement. Durability and environmental aspects are also addressed. Fiber addition may reduce chloride ingress in CAC, although long-term durability data remain limited. The use of coral aggregate must be balanced with the need to protect coral reefs. Finally, key knowledge gaps and future research directions are identified to support the sustainable application of FRCAC in marine infrastructure.

## 1. Introduction

The construction of artificial islands and reefs has become an important issue as the global demand for the exploitation of marine resources and protection of territorial waters increases [1,2]. However, islands far from the mainland often face a shortage of construction materials, especially traditional raw materials, such as river sand and gravel, for concrete production. Inland transportation of these materials is not only costly but also significantly increases carbon dioxide emissions and nonrenewable energy consumption. In addition, inland transportation can be subject to unpredictable weather conditions, which can cause delays in the construction schedule [3]. Therefore, exploring and utilizing locally available marine resources, such as coral reef debris (i.e., coral aggregates), has become an effective way to address the shortage of construction materials on remote islands.

Coral reefs are particularly abundant globally, especially on islands in tropical and subtropical regions, such as the South China Sea islands, the Maldives archipelago, and the Indonesian archipelago [4]. As a result of natural weathering and erosion, a large amount of loose, unconsolidated coral rock debris has accumulated on beaches [5]. Additionally, a large amount of coral debris is generated by marine activities, such as oil well construction and channel dredging [6]. These waste coral debris can be converted into coral aggregates suitable for concrete production after crushing, screening, and processing. Obtaining coral rubble for concrete preparation from waste coral rubble produced through natural weathering, channel excavation, and harbor dredging will not damage the natural ecosystem and will reduce the occupation of space on deserted islands by waste coral rubble [7].

Concrete produced from coral aggregates (CAs) is referred to as coral aggregate concrete (CAC), with seawater used as both the mixing and curing agent. The United States pioneered the study and utilization of coral aggregate concrete. During World War II, coral concrete was extensively used to build airports, highways, and buildings on islands in the Western Pacific, such as Midway, Kwajalein, Eniwetok, Bikini, Johnston, Wake, Saipan, and Guam [8,9,10,11,12]. Some of these structures are still in use today. In the 1950s and the 1960s, the U.S. Naval Civil Engineering Laboratory conducted extensive testing and evaluation of CAC structures, publishing reports on standard requirements for coral aggregate mining and processing, CAC mix proportions, and mixing standards [13,14]. In 1991, Ehlert [15] conducted a field investigation of three coral aggregate concrete structures on the Pacific Bikini atoll. The results showed that the compressive strength of the CAC structures, which was 24.7 MPa at 28 days, increased to 38.6 MPa after 11 to 16 years, a growth of 56%. China began exploring the use of coral aggregates in concrete in the 1970s, representing a relatively late stage in the development of this technology. In recent years, driven by the increasing demand for island and reef construction, both research interest and practical applications of coral aggregate concrete have grown rapidly [16]. Early investigations primarily focused on its basic mechanical properties. However, under the high-temperature, high-humidity, and high-salinity conditions characteristic of the South China Sea, early coral concrete mixtures generally exhibited compressive strengths below C30, failing to meet engineering requirements [17]. Fiber materials, characterized by their low density, high strength, fatigue resistance, and corrosion resistance, have been widely adopted to enhance the performance of cement-based composites. Consequently, the incorporation of fibers into coral aggregate concrete has emerged as an effective strategy to overcome its inherent performance limitations and become a key focus of current research.

Several recent review papers have provided comprehensive discussions on coral aggregate concrete (CAC), including its material properties, durability performance, and modification techniques, as well as Fiber Reinforced Polymer rebar (FRP) reinforced coral concrete from material and structural perspectives [5,18,19,20]. However, a systematic review specifically focusing on fiber-reinforced coral aggregate concrete (FRCAC) remains limited. Moreover, existing experimental studies on FRCAC are fragmented, with considerable variations in fiber types, mix proportions, testing methods, and performance evaluation criteria. In particular, the effects of different fibers on mechanical properties, dynamic behavior, durability, and performance under complex loading conditions have not yet been comprehensively synthesized. Therefore, this paper presents a comprehensive review of FRCAC, aiming to clarify fiber-strengthening mechanisms, identify current research gaps, and provide a structured reference for future experimental studies and engineering applications.

## 2. Constituent Materials of FRCAC

Fiber-reinforced coral aggregate concrete (FRCAC) is a multi-phase composite whose performance is strongly dependent on the properties of its constituent materials. The use of porous coral aggregates, marine mixing conditions, and various fiber reinforcements leads to material behaviors that differ markedly from those of conventional concrete. Understanding the characteristics of these constituents is therefore essential for interpreting the mechanical and durability performance of FRCAC. This section reviews the fundamental properties of coral aggregates, fibers, and seawater-related materials used in FRCAC.

### 2.1. Coral Aggregates

**Figure 1:**
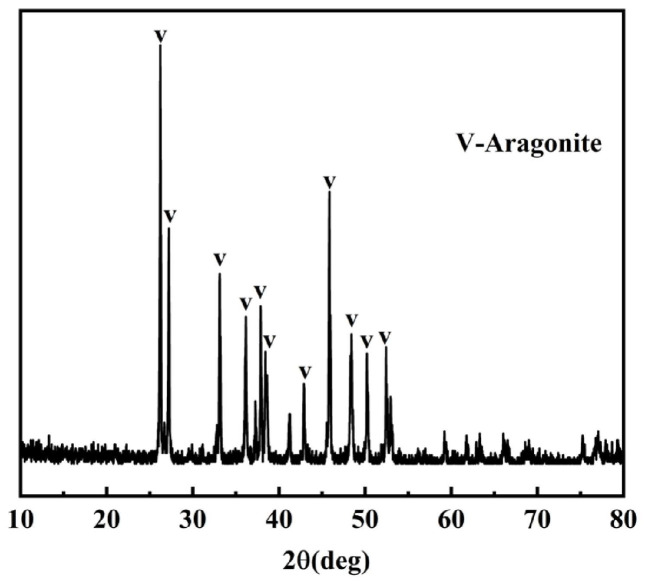
XRD pattern of coral aggregate [22].

**Figure 2:**
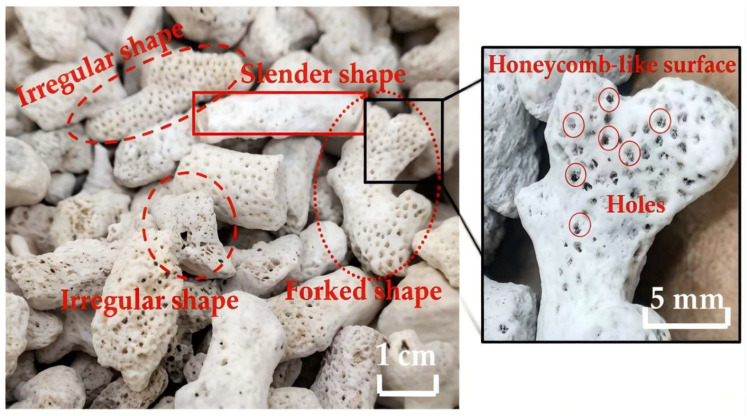
Morphology of coral aggregate [24].

Coral aggregates are derived from coral reefs, which are primarily composed of calcium carbonate (CaCO_3_), with a content of over 96% [21], and whose mineral composition is dominated by aragonite and calcite [22], as shown in Figure 1. These minerals do not easily react with alkali. Coral reefs exhibit remarkable diversity in their shapes, featuring distinctive branch-like and linear structures that account for 52.6% [23]. As shown in Figure 2, corals have different shapes, which may be elongated, forked, or irregular. In addition, there are many holes in coral, showing a honeycomb-like surface [24]. To enhance the tube compressive strength of coral aggregates, it is essential to optimize grading through crushing and screening processes.

The physico-mechanical properties of coral aggregates are affected by various factors, including coral species, degree of weathering, and mining depth. This results in a wide range of physico-mechanical properties. Table 1 and Table 2 present the physical properties of coral coarse and fine aggregates, respectively, as discussed in the literature. Coral coarse aggregates possess an apparent density of approximately 1800 kg/m^3^ and a bulk density of approximately 900 kg/m^3^ [25], classifying it as a natural lightweight aggregate. Its tube compressive strength is typically less than 3 MPa [26], which is significantly lower than that of commonly crushed stone aggregates (22–140 MPa). However, the tube compressive strength of high-quality staghorn coral reef stone has been observed to reach 6.8 MPa [27]. Coral aggregates possess a substantial degree of porosity, typically ranging from 47.3% to 55.6% [23], which leads to their low strength and high water absorption. Its pore structure is complex, with different pore morphologies on the inner and outer surfaces, including aligned macropores, circular pore clusters, and dense micropores on the outer surface. The internal structure consists of dense micropores, dendritic pores, circular pore clusters, anthill-like pores, and bread-like pores [28]. This complex pore structure enables coral aggregates to exhibit distinct pore permeability thresholds when compared to other soft-matter particle systems, such as volcanic rocks [29]. During the concrete mixing process, coral aggregates absorb a proportion of the mixing water, thereby altering the water–cement ratio surrounding the aggregate and impeding the formation of ‘blisters’ in the lower portion of the aggregate [27]. Concurrently, the absorbed water is released during the later stages of concrete hardening, thereby creating an ‘internal curing’ effect. This effect promotes cement hydration, reduces the porosity of the concrete, and results in a denser transition zone at the aggregate–mortar interface [30]. Consequently, it is imperative that coral aggregates be pre-wetted before the utilization of coral aggregate concrete.

In summary, coral aggregates possess surfaces that are both rough and porous, in addition to complex internal pore systems, resulting in high porosity and water absorption properties. These unique properties and processing methods present challenges and opportunities for coastal construction applications. Further investigation into the variability of its physico-mechanical properties is required to facilitate comprehensive understanding and optimization of its application in engineering projects. To regulate the application of coral aggregates in China, the relevant authorities implemented ‘Coral Aggregate for Concrete’ [35] and ‘Technical Specification for the Application of Coral Aggregate for Concrete’ [36] on 1 November 2020 with the objective of promoting the design, construction, and operation of offshore coral concrete towards systematization and standardization.

### 2.2. Fiber Types and Characteristics

**Figure 3:**
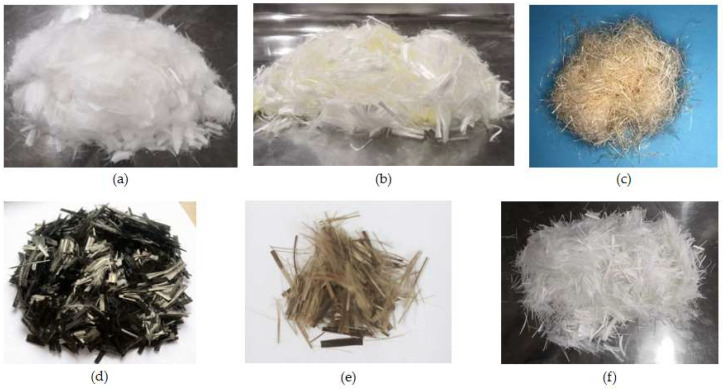
Morphology of fibers: (**a**) polypropylene fiber (PPF); (**b**) polyvinyl alcohol fiber (PVA); (**c**) sisal fiber (SF); (**d**)carbon fiber (CF); (**e**) basalt fiber (BF); (**f**) glass fiber (GF).

To improve the performance of coral aggregate concrete, it is advisable to incorporate fibers for toughening and strengthening. Different types of fibers have distinct characteristics. The seven types of fibers commonly used in coral concrete have their basic properties and shapes detailed in Table 3 and Figure 3.

Polypropylene fiber (PPF) is an organic fiber that possesses excellent dispersibility and chemical corrosion resistance and can enhance the strength of coral concrete. However, the incorporation of fly ash is required with PPF to achieve optimal results, as it improves the interfacial transition zone [43]. Polyvinyl alcohol fiber (PVA), another organic fiber, is renowned for its strong corrosion resistance and excellent bonding properties with concrete, and it can significantly improve the impact compression properties of coral concrete, including dynamic compressive strength, dynamic modulus of elasticity, and impact toughness [44]. Sisal fiber (SF), a natural organic fiber, possesses inherent strengths and toughness; however, it is susceptible to brittleness in alkaline environments. The addition of silica fume during production can mitigate the formation of calcium hydroxide, thereby enhancing durability [45]. Carbon fiber (CF), an inorganic fiber, is renowned for its exceptional strength, elastic modulus, and resistance to corrosion and high temperatures. Despite its relatively high cost, carbon fiber significantly improves the fracture strength of coral concrete, effectively preventing the formation of cracks [46]. Basalt fiber (BF) is an inorganic fiber with high strength, high elastic modulus, acid and alkali resistance, high temperature resistance, and good compatibility with cementitious materials [47]. They can enhance the mechanical properties of coral aggregate concrete, such as porosity and strength, and positively affect its triaxial mechanical properties. Glass fiber (GF), categorized as an inorganic fiber, exhibits elevated strength and exceptional alkali resistance, thereby effectively counteracting the erosion of alkaline substances within the hydration products of cement, thus enhancing the performance of coral aggregate concrete [48]. The synergy of these fibers leads to a substantial enhancement in the mechanical properties and durability of coral aggregate concrete, thereby expanding its application prospects in specialized environments, such as marine engineering.

### 2.3. Seawater as a Mixing Medium

**Figure 4:**
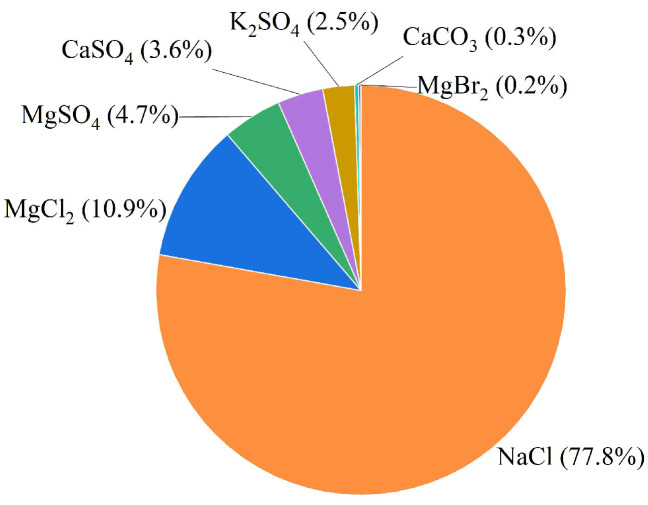
Major salt components of seawater.

Seawater is a complex natural body of water composed primarily of salts, minerals, and dissolved gases with a composition that varies significantly between different seas. The salinity of seawater is highest in the South China Sea, with an average of 34.15%, and lowest in the East Sea, followed by the Yellow Sea and the Bohai Sea [49]. The predominant ions in seawater include Na^+^, K^+^, Ca^2+^, Mg^2+^, Cl^−^, and SO_4_^2−^, among others, which collectively form intricate electrolyte solutions [50]. Based on representative compositional data reported in reference [50], Figure 4 illustrates the typical salt composition of seawater.

The disparity in the performance of seawater- and freshwater-mixed concrete can be attributed to the presence of inorganic salts in seawater. Seawater-mixed plain concrete appears to incorporate inorganic salt admixtures, which have been observed to enhance early strength while exerting minimal influence on durability [51]. The Ministry of Transport of China stipulates that in areas where freshwater resources are scarce, seawater can be used to mix vegetal concrete, and the water–cement ratio can be lowered by 0.05 when there is a requirement for freezing resistance. When seawater is used to mix coral aggregate concrete, the hardening of the cement set and the microstructure differ from those of conventional concrete. The presence of ions in seawater has been shown to accelerate the hydration of cement and increase the degree of hydration, resulting in the rapid development of strength in the early stages and subsequent slow development in later stages [52]. This has led to the observation that the strength of concrete can reach up to 80% of its 28-day strength by 7 days. Concurrently, the presence of Ca^2+^ and Cl^−^ has been observed to exert an adverse influence on C-S-H gel density, thereby impeding the advancement of strength during the final stages of development [53]. A long-term field exposure study conducted on a marine island project reported that seawater-mixed concrete, including mixtures using coral-derived aggregates, showed no measurable strength loss or corrosion-related deterioration after up to 19 years of marine exposure, demonstrating the long-term feasibility of seawater as a mixing medium in such environments [54].

In the context of concrete comprising sea sand and coral reef aggregate as aggregates, with seawater serving as the mixing medium, chloride (Cl^−^), magnesium ions (Mg^2+^), and sulfate (SO_4_^2−^) ions are directly generated as reaction products within the concrete matrix. This phenomenon underscores the necessity of prioritizing durability concerns in concrete-related research and applications. Experimental evidence has demonstrated that chloride ions exacerbate the corrosion of steel reinforcement in coral aggregate concrete, resulting in a corrosion rate that can be up to twice that of normal aggregate concrete [55,56,57]. Several preventive methods have been proposed, including increasing the thickness of the protective layer [58], using corrosion-resistant reinforcement [59,60], reducing chloride ion permeability [61], and using rust inhibitors [50].

Therefore, seawater-mixed concrete can meet strength requirements. The use of corrosion-resistant reinforcing materials, such as stainless steel, epoxy-coated steel, or FRP bars, in conjunction with seawater concrete, reduces the impact of chloride ions on structural durability. The use of seawater in island construction is feasible and economical.

## 3. Mechanical Properties of FRCAC

Different fiber types have been introduced into coral aggregate concrete to enhance its basic mechanical properties. However, owing to the distinct mechanical characteristics of fibers and their interaction mechanisms with the cementitious matrix, the strengthening effects reported in the literature vary considerably. Therefore, this section systematically reviews experimental results on the compressive, splitting tensile, flexural, and elastic properties of fiber-reinforced coral aggregate concrete under uniaxial loading, aiming to clarify the general trends and underlying mechanisms.

### 3.1. Compressive Strength

The compressive strength of concrete is a pivotal parameter in the structural design, quality control, economic efficiency, and cost management of concrete structures. The mechanical properties of FRCAC are closely linked to the performance of the concrete matrix but primarily determined by the type, dosage, geometry, and mechanical properties of the fibers.

#### 3.1.1. Overall Trends

**Figure 5:**
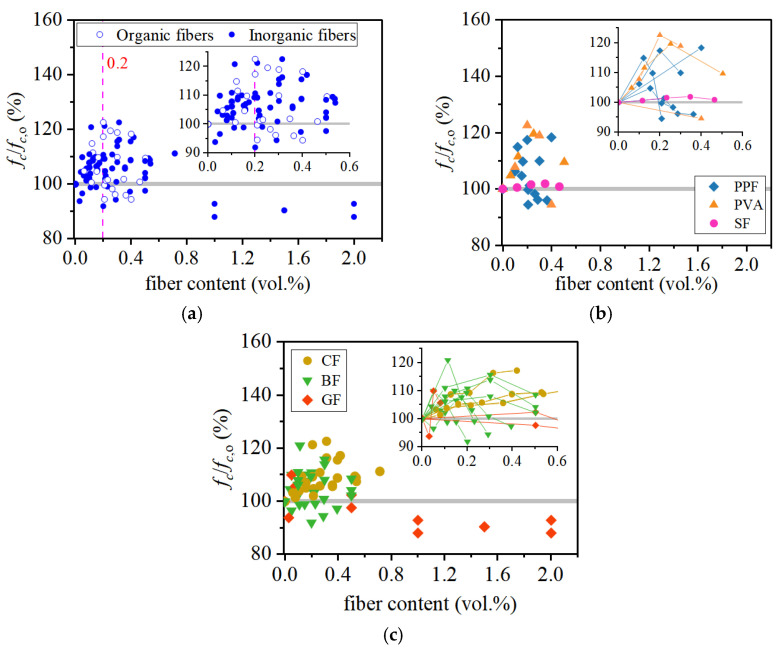
The compression behavior of FRCAC: (**a**) all fiber types:organic fibers [42,62,63,64,65,66], inorganic fibers [25,28,40,41,42,63,67,68]; (**b**) organic fibers: PPF [42,62,63], PVA [62,64,65], SF [66]; (**c**) inorganic fibers: CF [28,40,63], BF [25,41,42,63,67], GF [42,68].

As illustrated in Figure 5, many researchers have conducted experimental investigations of the compressive behavior of FRCAC incorporating various fibers. The fibers used in FRCAC include polypropylene fibers (PPF) [42,62,63], polyvinyl alcohol fibers (PVA) [62,64,65], sisal fibers (SF) [66], carbon fibers (CF) [28,40,63], basalt fibers (BF) [25,41,42,63,67], and glass fibers (GF) [42,68], with fiber lengths ranging from 6 to 36 mm. Based on material characteristics, PPF, PVA, and SF are generally classified as organic fibers, whereas BF, GF, and CF are considered inorganic fibers.

As summarized in Figure 5a, the compressive strength performance of FRCAC is presented in terms of its relative strength compared with coral aggregate concrete (CAC) over a wide range of fiber volume fractions. The reported 28-day compressive strength of FRCAC generally ranges from approximately 21.0 to 55.8 MPa, with most fiber volume fractions below 2.0 vol.%. Under optimized fiber types and dosages, the maximum reported enhancement in compressive strength can reach 23%. However, the majority of results fall within a more moderate improvement range. The majority of data points are concentrated at fiber contents lower than about 0.20 vol.%, where most FRCAC specimens exhibit compressive strengths comparable to or slightly higher than those of CAC. As the fiber dosage further increases, the scatter of experimental results becomes markedly larger, with both strength enhancement and strength reduction reported.

Microscopic observations reported in the literature indicate that this behavior is closely associated with fiber dispersion and fiber–matrix interactions. Well-dispersed fibers can partially restrain crack propagation under compressive loading, whereas excessive fiber contents often lead to fiber agglomeration, increased porosity, and interfacial defects, which offset the potential reinforcing benefits [28,41,42]. Therefore, fiber dosage alone is insufficient to explain the observed variability in compressive strength, and further analysis from the perspective of fiber characteristics is required.

#### 3.1.2. Effect of Fiber Category

The influence of fiber category on compressive strength is further illustrated in Figure 5b,c, which distinguish the responses of FRCAC reinforced with organic and inorganic fibers, respectively. For organic fibers (PPF, PVA, and SF), most data points cluster around a relative compressive strength close to that of CAC, suggesting a generally modest enhancement with pronounced scatter. In contrast, inorganic fibers—particularly carbon fibers—exhibit a wider distribution and a higher potential for compressive strength improvement, although significant variability is also evident, reflecting their fundamentally different stiffness and interfacial interactions with the porous coral matrix. These observations indicate that fiber classification alone is insufficient to predict the compressive performance of FRCAC. The large scatter within each fiber category suggests that more fundamental parameters related to fiber geometry, dispersion, and matrix quality must be considered.

#### 3.1.3. Effect of Fiber Geometry

When the influence of fiber geometry on the compressive behavior of FRCAC is examined in greater detail, basalt-fiber-reinforced CAC is found to exhibit a clear length-dependent response. Adequately long and well-dispersed BF can stabilize compressive behavior by restraining lateral dilation and delaying crack coalescence, whereas short or poorly dispersed BF may even reduce compressive strength by acting as local heterogeneities within the porous coral matrix [67]. Similar length-dependent trends have been reported for alkali-resistant glass fibers in CAC, where longer fibers provide more effective crack bridging and load sharing than shorter ones at comparable fiber contents [68]. In addition, PVA fibers in coral-cement-based materials also show a geometry-dependent response, with an intermediate length-to-diameter ratio (L/D) yielding optimal compressive performance, while excessive fiber length leads to fiber entanglement and increased porosity [69].

**Figure 6:**
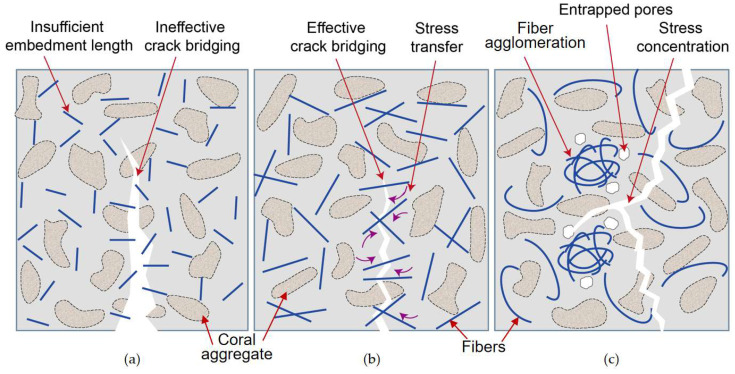
Schematic illustration of the influence of fiber geometry on crack-bridging efficiency in FRCAC: (**a**) short fibers; (**b**) fibers with optimal length; (**c**) overlong fibers.

These consistent observations indicate that fiber length and aspect ratio largely control whether fibers can be effectively mobilized in the highly porous coral matrix. As schematically illustrated in Figure 6, sufficiently long and well-dispersed fibers form a crack-bridging network and contribute to stress transfer under compression, whereas short or poorly dispersed fibers tend to behave as inert inclusions and promote interfacial defects and pore accumulation.

#### 3.1.4. Effect of Fiber Stiffness

**Figure 7:**
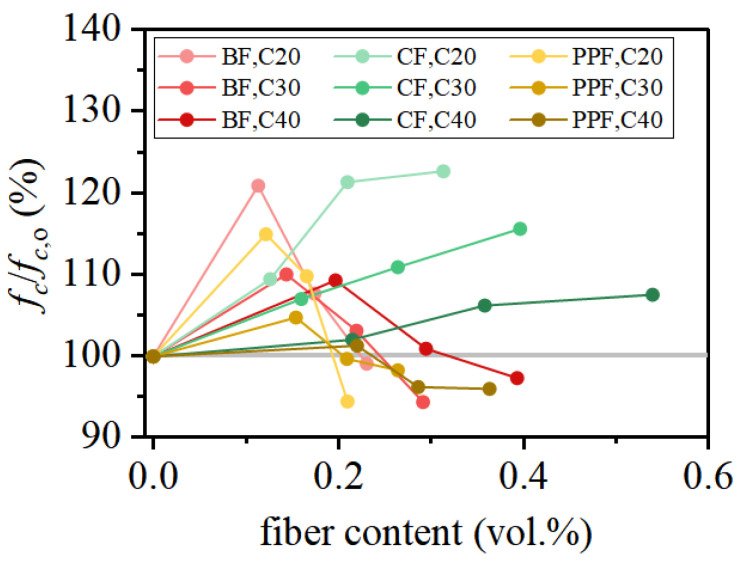
Effect of fiber stiffness on the compressive strength of FRCAC under comparable test conditions [63].

Fiber stiffness is another key factor governing the compressive performance of FRCAC. For carbon-fiber-reinforced coral aggregate concrete (CFRCAC), the strengthening effect gradually diminishes when the CF content exceeds approximately 0.2 vol.%, although the compressive strength generally remains higher than that of CAC. Deng et al. [63] reported that at comparable strength levels, CF—owing to its high elastic modulus—provides more effective compressive reinforcement than low-modulus fibers, such as BF and PPF, as illustrated in Figure 7. The reinforcement effect of low-modulus synthetic fibers is less pronounced, particularly at higher dosages. This phenomenon can be attributed to the weaker bond strength, inferior fiber bridging capability, and increased porosity within the concrete matrix [70,71]. High-modulus fibers are capable of redistributing localized internal stresses and reducing stress concentrations, thereby suppressing localized failure [72].

#### 3.1.5. Effect of Curing Age and Hydration Development

**Figure 8:**
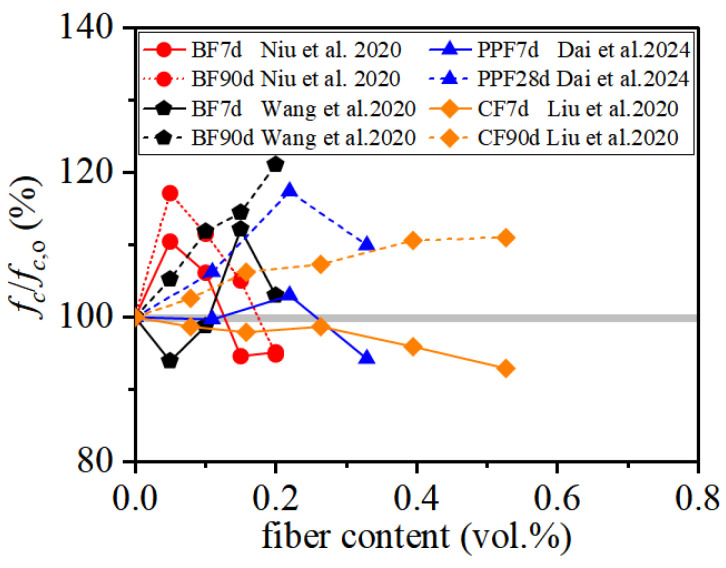
Compressive strength of FRCAC at different curing ages [25,28,41,42].

Beyond fiber type and dosage, curing age is a critical variable because the hydration progress and interfacial bond development of the porous coral-based matrix are strongly time-dependent, thereby governing the extent to which fibers can be effectively mobilized. As shown in Figure 8, increasing CF content may slightly reduce early-age compressive strength while enhancing long-term strength, which has been attributed to delayed cement hydration caused by CF incorporation [28]. For BF-reinforced coral aggregate concrete (BFRCAC), some studies report an initial reduction in early-age strength followed by strength recovery and improvement at later ages due to pore filling and improved fiber–matrix bonding with ongoing hydration [41]. Conversely, Niu et al. [25] observed an initial increase followed by a decrease in both early-age and long-term compressive strength with increasing BF dosage. Such discrepancies highlight that variations in fiber properties, including fiber length, diameter, tensile strength, and elastic modulus, can significantly affect fiber dispersion, interfacial bonding, and the optimal fiber dosage, leading to diverse compressive strength development trends.

#### 3.1.6. Summary of Compressive Behavior

In summary, the incorporation of an appropriate amount of fibers can moderately enhance the compressive strength of FRCAC, particularly when fibers with high elastic modulus and suitable geometry are employed. Low-modulus fibers generally exhibit a weaker strengthening effect, especially at higher dosages, where fiber agglomeration and pore entrapment may offset their reinforcing contribution. More importantly, the literature does not reveal a unified trend with respect to fiber type alone, indicating that compressive performance is governed by a coupled effect of fiber stiffness, fiber geometry, matrix maturity, and test conditions. Variations in curing age, water-to-binder ratio, specimen geometry, and loading configuration further modulate the extent to which fiber reinforcement can be effectively mobilized, thereby giving rise to the large scatter observed in multi-source datasets.

### 3.2. Splitting Tensile Strength

**Figure 9:**
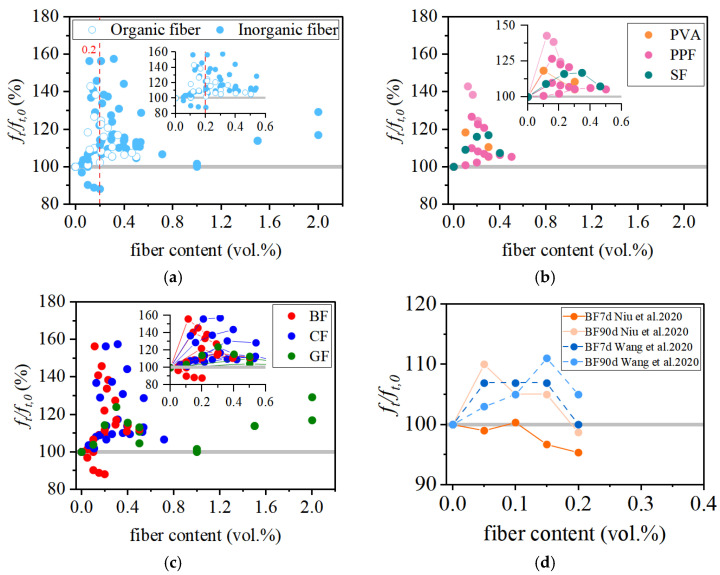
The tensile behavior of FRCAC: (**a**) all fiber types:organic fiber [62,63,66,73], inorganic fiber [25,28,41,63,68,73]; (**b**) organic fiber: PVA [62], PPF [63,73], SF [66]; (**c**) inorganic fiber: BF [25,41,63,73], CF [28,63,73], GF [68]; (**d**) development of splitting tensile strength [25,41].

The tensile performance of fiber-reinforced coral aggregate concrete (FRCAC) is commonly evaluated using splitting tensile tests, which are typically conducted alongside compressive tests. As shown in Figure 9a, the splitting tensile strength of FRCAC incorporating various fiber types and dosages is generally higher than that of CAC, indicating a clear tensile strengthening effect. Compared with compressive strength, the enhancement in splitting tensile strength exhibits less scatter, reflecting the effectiveness of fibers in controlling crack initiation and propagation under tensile loading [41,73]. Despite the variability among different studies, the compiled results suggest that fiber reinforcement can lead to a substantial improvement in splitting tensile strength, with the maximum reported increase reaching up to 58% relative to CAC, although most results remain within a moderate enhancement range.

Figure 9b,c further distinguish the effects of organic and inorganic fibers on the splitting tensile strength of FRCAC, respectively. For organic fibers, including PVA [62], PPF [63,73], and SF [66], most reported results demonstrate a noticeable improvement in splitting tensile strength. This enhancement is primarily attributed to effective crack bridging and suppression of crack opening under tensile stress. For inorganic fibers, CF [28,63,73] and GF [68] also exhibit a positive tensile strengthening effect, with CF generally showing the most pronounced improvement among the investigated fibers. This behavior is attributed to the high elastic modulus of CF, which enables early stress sharing upon microcrack initiation within the matrix, whereas low-modulus fibers tend to contribute to load transfer primarily after matrix cracking, resulting in a less pronounced tensile enhancement [73].

For basalt fibers (BF) [25,41,63,73], the reported splitting tensile strength results show considerable variability, with both strength enhancement and reduction observed among different studies. Data-driven analysis by Sun et al. [74] demonstrates that this variability is primarily governed by matrix maturity. Their Shapley additive explanations (SHAP) analysis identifies curing age and the water-to-binder ratio as the dominant controlling variables for the splitting tensile strength of BFRCAC [74]. In addition, experimental studies show that BF length is a critical geometric parameter, as only sufficiently long and well-dispersed fibers are able to effectively bridge microcracks and restrain crack opening [67]. Consequently, BF can enhance splitting tensile strength when incorporated into a dense and mature matrix with suitable fiber geometry, whereas in weak or highly porous matrices, its reinforcing efficiency is greatly reduced and may even become negative [75]. Moreover, variations in specimen geometry and splitting-test configuration among different studies also contribute to the scatter of reported tensile strength, further modulating the apparent efficiency of fiber bridging.

It is noteworthy that fibers such as SF and GF exhibit only limited enhancement in compressive strength, whereas they provide a more pronounced improvement in splitting tensile strength. This behavior is primarily related to their mechanical characteristics and deformation behavior within the concrete matrix. Owing to their relatively lower elastic modulus and slender geometry, these fibers contribute little to direct load-bearing capacity under compression but are readily mobilized in tension once microcracks initiate. Consequently, their dominant role in fiber-reinforced cementitious composites, including coral aggregate concrete, is to bridge cracks and restrain crack propagation rather than to directly carry compressive stresses.

Similar to compressive strength, fiber incorporation also influences the evolution of splitting tensile damage in coral aggregate concrete. In particular, basalt fibers (BF) exhibit a more pronounced effect on late-stage splitting tensile performance than in early stages, as illustrated in Figure 9d. Experimental results reported in [41] indicate that during the early curing stage (within 7 days), the incorporation of 0.05% and 0.10% BF led to a slight reduction in the splitting tensile strength of coral aggregate concrete. In contrast, a BF content of 0.15% resulted in the most significant enhancement in long-term (90-day) splitting tensile strength [73]. These findings suggest that the contribution of BF to tensile resistance is closely associated with the development of fiber–matrix bonding and the progressive densification of the cementitious matrix with curing age.

In summary, fiber reinforcement effectively improves the splitting tensile strength of coral aggregate concrete by enhancing crack control and resistance to crack propagation. Compared with compressive strength, the tensile performance of FRCAC is generally more responsive to fiber incorporation, with high-modulus fibers showing superior enhancement and low-modulus fibers mainly contributing through crack-bridging mechanisms. Although the tensile response of basalt-fiber-reinforced concrete exhibits noticeable scatter, fiber reinforcement overall plays a crucial role in improving the tensile capacity and cracking resistance of coral concrete.

### 3.3. Flexural Strength

**Figure 10:**
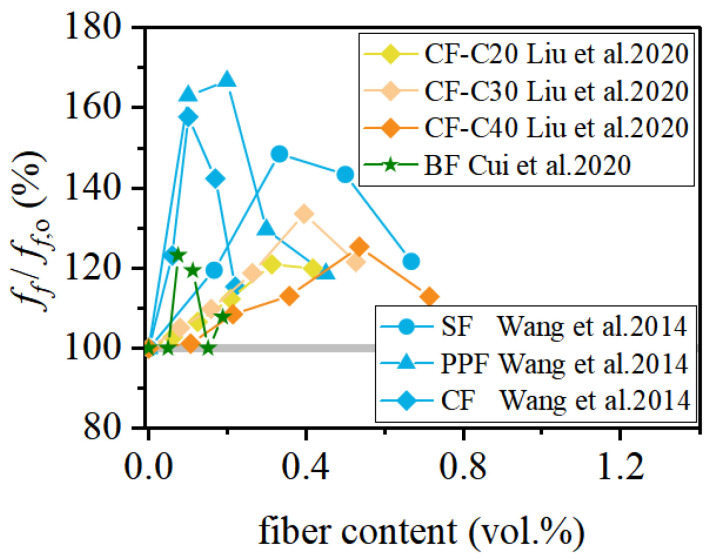
The flexural behavior of FRCAC [37,40,66,75,76].

Table 4 summarizes recent experimental investigations on the flexural strength of FRCAC. Figure 10 compares the relative flexural strengths of FRCAC and CAC. The fiber types examined mainly include polypropylene fibers (PPF), basalt fibers (BF), carbon fibers (CF), and sisal fibers (SF), with fiber volume contents generally ranging from 0% to 0.7%. The reported flexural strength of FRCAC varies from 2.6 to 6.72 MPa and consistently exceeds that of CAC. Within an appropriate dosage range, increasing fiber content generally leads to improved flexural performance of coral concrete. This enhancement is primarily attributed to the ability of fibers to bridge tensile cracks and delay crack propagation in the tension zone under bending, which is consistent with the mechanisms discussed in the splitting tensile strength section.

Among the investigated fibers, polypropylene fibers (PPF) exhibit a particularly pronounced effect on flexural strength. For example, when the PPF content increased from 0% to 0.2%, the flexural strength of coral concrete increased by 66.67%, reaching 4.5 MPa [37]. The particularly pronounced enhancement of flexural strength observed in polypropylene-fiber-reinforced coral aggregate concrete can be attributed to the crack-dominated nature of flexural failure. Under bending, tensile stresses develop in the tension zone, where failure is governed by crack initiation and propagation rather than by compressive load-bearing capacity. Owing to their small diameter, high fiber count, and uniform dispersion, polypropylene fibers form a dense fiber network that effectively restrains the initiation and growth of microcracks in the tensile zone. Although PPF possesses a relatively low elastic modulus, it becomes progressively mobilized after crack initiation, providing stable crack-bridging resistance and delaying crack localization. In addition, the favorable interfacial interaction between PPF and the porous coral concrete matrix enhances energy dissipation during fiber pull-out, thereby contributing to the significant improvement in flexural strength and deformation capacity.

Carbon fibers (CF) can also effectively enhance the flexural performance of coral concrete; however, their reinforcing efficiency strongly depends on the strength level of the concrete matrix and fiber geometry. In particular, CF with a high aspect ratio generally demonstrate superior flexural strengthening capability [40,76]. In contrast, basalt fibers (BF) tend to exhibit a less pronounced improvement in flexural strength compared with other fiber types, which may be associated with differences in fiber stiffness, dispersion, and fiber–matrix interfacial behavior [75]. Sisal fibers (SF) show a moderate enhancement in flexural strength, mainly through crack-bridging mechanisms, but available data remain limited.

Overall, existing studies indicate that fiber reinforcement can significantly improve the flexural behavior of coral aggregate concrete. However, experimental data on the flexural response of FRCAC are still relatively scarce. Further investigations are therefore required to clarify fiber action mechanisms under bending, optimize fiber parameters, and support the reliable application of fiber-reinforced coral concrete in flexural–critical structural members.

### 3.4. Elastic Modulus

**Figure 11:**
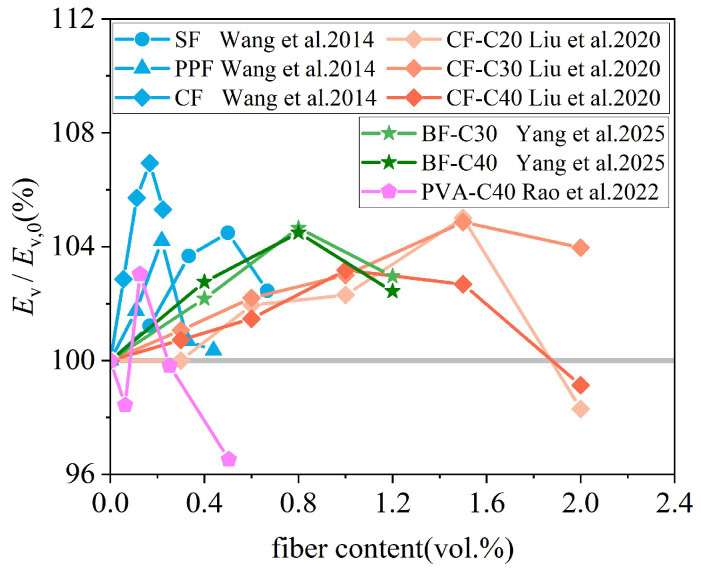
The elastic modulus of FRCAC [37,65,66,76,78].

Figure 11 summarizes recent experimental results on the relationship between the fiber content and the elastic modulus of FRCAC, covering several fiber types, including SF [66], PPF [37], CF [76,77], BF [78], and PVA [65]. Overall, fiber incorporation can lead to a moderate enhancement in the elastic modulus of coral concrete under appropriate conditions. However, the magnitude of this improvement remains relatively limited, with reported increases generally ranging from 0% to 7% and averaging approximately 4%.

As illustrated in Figure 11, the elastic modulus of FRCAC typically exhibits a non-monotonic trend with increasing fiber content, characterized by an initial increase followed by a gradual decrease. This behavior suggests the existence of an optimal fiber dosage, which varies among different fiber types. When the fiber content exceeds this optimal level, the elastic modulus of FRCAC may even decrease to values lower than those of fiber-free coral aggregate concrete [65]. With optimal fiber content, fibers are more uniformly dispersed within the cementitious matrix, forming a relatively stable network that helps restrain microcrack development and promotes more uniform stress distribution under loading, thereby contributing to a slight increase in elastic stiffness. This is mainly because the elastic modulus is governed by the matrix and aggregate stiffness at small strain levels, where fiber’s contribution remains limited. In contrast, excessive fiber addition tends to reduce workability and increase porosity, which offsets the reinforcing effect and leads to a reduction in the elastic modulus. In addition, existing studies indicate that at comparable optimal fiber contents, lower-strength-grade coral concrete generally exhibits a higher relative increase in elastic modulus than higher-strength concrete [77,78].

Despite these observations, the available data on the elastic modulus of FRCAC remain limited. Further studies are therefore required to clarify the combined effects of concrete strength, fiber dosage, and fiber type on elastic modulus development.

### 3.5. Summary of Basic Mechanical Properties

In summary, the incorporation of different fiber types enhances the basic mechanical properties of coral aggregate concrete to varying extents, and each fiber system exhibits a distinct optimal dosage range. Overall, fiber reinforcement provides the most pronounced improvement in splitting tensile and flexural strengths through crack-bridging and crack-control mechanisms, whereas its influence on compressive strength and elastic modulus remains relatively limited. Based on the experimental evidence discussed above, Table 5 provides an engineering-oriented comparison of the typical performance benefits and limitations of different fiber types, aiming to facilitate material selection for practical applications of FRCAC. It should be noted that the mechanical property data reviewed in this section are predominantly obtained from short-term laboratory tests on small-scale specimens. The age-dependent evolution of fiber reinforcement effects and the mechanical performance of FRCAC under long-term service conditions in real structures remain largely unexplored.

From an engineering perspective, different fiber types exhibit distinct advantages and limitations, and the selection of fiber type and dosage should therefore be guided by specific performance requirements and service conditions. Although recent studies have suggested that hybrid fiber systems may offer synergistic benefits by combining the complementary roles of different fibers, such approaches typically involve more complex interaction mechanisms and optimization strategies [79,80]. Consequently, the present review primarily synthesizes the mechanical performance of coral aggregate concrete reinforced with single fiber types, while hybrid fiber systems are only briefly referenced as a promising direction for future research.

Moreover, due to the limited number of available experimental studies and variations in material properties, mix proportions, and testing protocols, reported optimal fiber dosages and strengthening effects are not always consistent. Further systematic experimental investigations, together with complementary numerical analyses, are therefore required to clarify fiber reinforcement mechanisms and to establish more reliable design guidelines for FRCAC.

## 4. Dynamic Mechanical Properties

As marine resource development and island and reef construction advance, greater emphasis is placed on the dynamic mechanical properties of construction materials. In particular, materials used in special environments, such as islands and reefs in the South China Sea, must exhibit adequate static mechanical properties and the capacity to resist dynamic loads, including impacts and explosions. Impact resistance is an important performance indicator of concrete used in civil engineering [81]. In actual projects, many concrete components are subjected to low-speed impact loads, such as pavements, breakwaters, and precast concrete piles. Currently, there are two main experimental methods to study the impact resistance of FRCAC: the drop-weight impact test and the Split Hopkinson Pressure Bar (SHPB) test.

### 4.1. The Drop-Weight Impact Test

**Figure 12:**
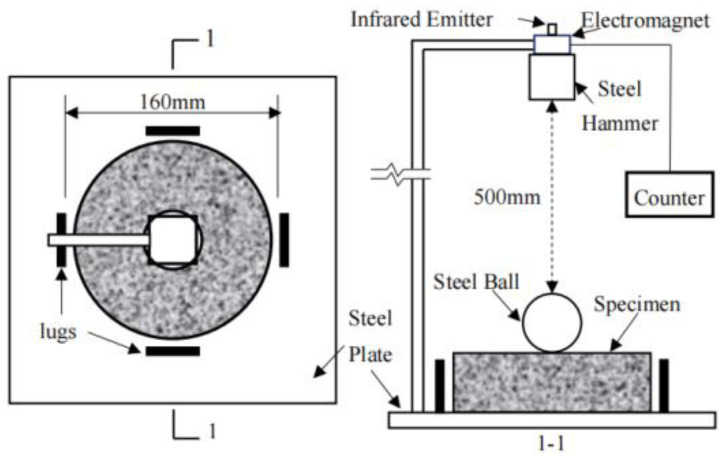
Details of the drop-weight impact test setup [82].

The drop-weight impact test is a widely used method for evaluating the impact resistance of concrete and composite materials. In this test, a weight is dropped from a predetermined height onto a specimen, subjecting it to dynamic loading. The energy absorbed by the specimen during impact is measured, providing valuable insight into its toughness and ability to withstand sudden dynamic forces. The drop-weight impact test setup is shown in Figure 12.

**Figure 13:**
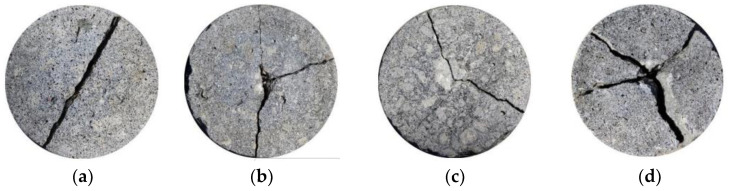
The failure patterns of part of CFRCAC specimens: (**a**) CAC; (**b**) CAC with 0.6%CF; (**c**) CAC with 1.5%CF; (**d**) CAC with 2.0%CF [82].

**Figure 14:**
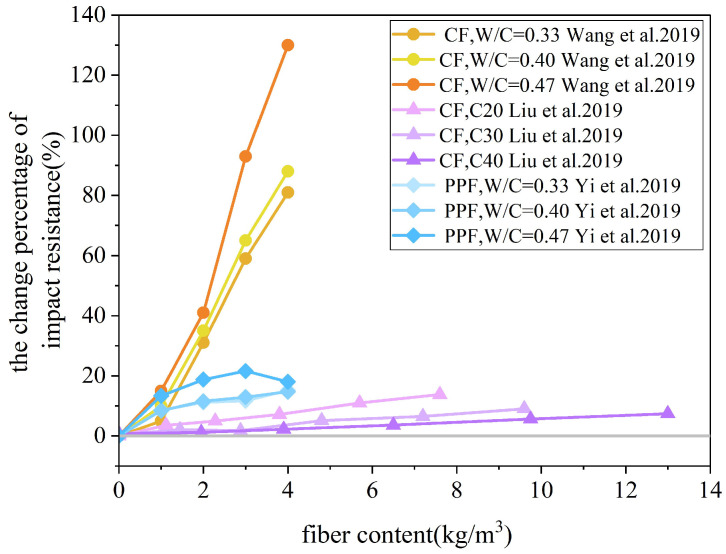
Impact resistance changes in FRCAC [82,83,84].

Studies have demonstrated that incorporating fibers can effectively dissipate the energy of coral aggregate concrete under impact conditions. After cracks appeared in the sample, the fibers spanning the failure section could still withstand the impact energy, thereby preventing further propagation of the initial crack and enhancing the impact resistance of the coral concrete. The impact failure diagrams of the CFRCAC specimens are illustrated in Figure 13, whereas the variations in the impact resistance of FRCAC at different water–cement ratios are depicted in Figure 14. Liu et al. [82] and Wang et al. [83] investigated the impact resistance of CFRCAC; perhaps owing to the differing experimental conditions, their experimental data exhibited some inconsistencies, although the observed patterns remained consistent. The carbon fiber coral concrete specimens exhibited significant ductile failure characteristics under impact loads. Within a certain range, an increase in the amount of carbon fiber corresponded to an enhancement in the impact resistance of coral concrete. Furthermore, Liu et al. [82] established that the two-parameter Weibull distribution theory can adequately represent the results of impact tests. However, the impact resistance of the samples with added PPF did not monotonically increase with the amount added. Excessive PPFs are not easily dispersed uniformly in the coral concrete matrix, leading to an increase in porosity and defects in the interfacial transition zone, which reduces the strength of the coral concrete and affects the bonding performance between the fibers and the paste, preventing the full enhancement of impact resistance [84].

### 4.2. The Split Hopkinson Pressure Bar (SHPB) Test

**Figure 15:**
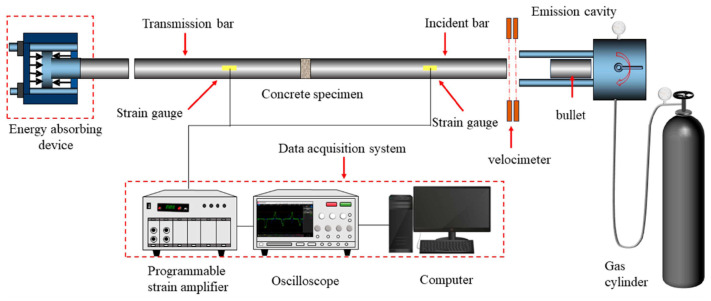
Schematic diagram of Split Hopkinson Pressure Bar (SHPB) test setup [85].

The Split Hopkinson Pressure Bar (SHPB) test has been widely adopted to investigate the mechanical behavior of concrete materials under high-strain-rate loading conditions. Compared with drop-weight impact testing, the SHPB technique enables more controlled and repeatable high-strain-rate loading, offering improved accuracy in characterizing dynamic compressive behavior. The experimental setup and fundamental principles of the SHPB system are illustrated in Figure 15. Existing studies consistently show that fiber reinforcement can significantly enhance the impact resistance and energy absorption capacity of coral aggregate concrete (CAC) under SHPB loading.

**Figure 16:**
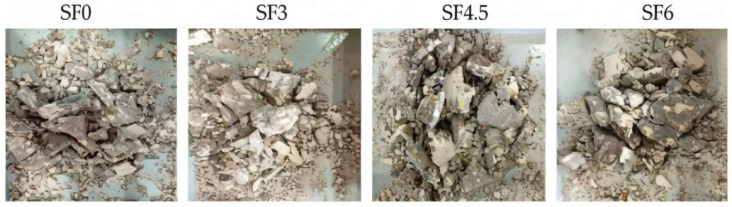
Impact compression failure diagram [85].

Experimental evidence indicates that natural fibers (e.g., SF [86,87]), synthetic fibers (e.g., PVA [88]), and hybrid fiber systems [89] all contribute to improved structural integrity and reduced brittle fragmentation of CAC at high strain rates. As illustrated by the representative failure modes in Figure 16, fiber-reinforced specimens exhibit less severe fragmentation and more cohesive failure patterns than CAC under SHPB impact compression, indicating that fibers effectively restrain crack propagation and delay catastrophic disintegration under rapid loading. Within typical strain-rate ranges of approximately 40–180 s^−1^, FRCAC generally exhibits higher dynamic compressive strength, peak strain, and energy absorption capacity than CAC. In addition, hybrid fiber systems combining fibers with different stiffness and ductility show enhanced impact toughness and more stable post-peak behavior [89]. Under marine semi-submerged conditions, SHPB tests further indicate that sisal fibers primarily contribute to mitigating impact-induced fragmentation and maintaining post-peak energy absorption after long-term seawater erosion, while their effect on peak dynamic compressive strength remains limited [85].

**Figure 17:**
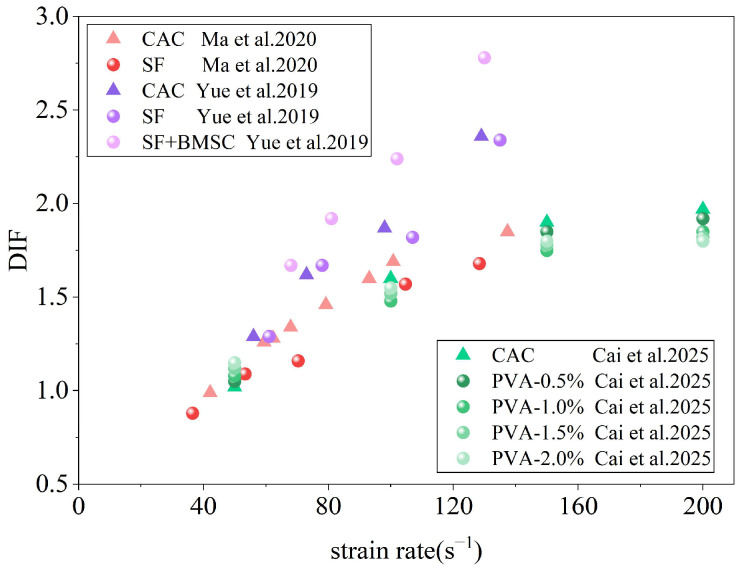
Relationship between impact compression DIF and strain rate [86,87,88].

Figure 17 shows that the dynamic increase factor (DIF) of coral aggregte concrete (CAC) increases with increasing strain rate, confirming a pronounced strain-rate effect. For mixtures containing only sisal fibers (SF), the DIF values are comparable to or slightly lower than those of the corresponding plain CAC, indicating that sisal fiber addition alone does not enhance the apparent strain-rate sensitivity. In contrast, the combined system incorporating sisal fibers and basic magnesium sulfate cement (BMSC) exhibits a clear upward shift in the DIF–strain-rate relationship, whereas PVA-reinforced CAC does not show a monotonic increase in DIF with fiber dosage.

### 4.3. Summary of Dynamic Mechanical Properties

In summary, both the drop-weight impact test and the Split Hopkinson Pressure Bar (SHPB) test are essential methods for evaluating the dynamic mechanical properties of fiber-reinforced coral aggregate concrete. The drop-weight impact test provides valuable data on the material’s performance under low-strain-rate impact conditions, which are representative of typical environmental and accidental loads. The inclusion of fibers significantly enhances the impact resistance and energy absorption of FRCAC, improving its ability to withstand sudden dynamic forces.

On the other hand, the SHPB test offers a more comprehensive analysis of the material’s behavior under high-strain-rate loading, such as that encountered in extreme events, like explosions or seismic activities. This test has shown that fiber reinforcement not only improves energy dissipation but also increases the material’s resistance to high-velocity impacts. Both tests highlight the importance of fiber reinforcement in improving the dynamic properties of FRCAC, with fiber type, content, and distribution playing critical roles in performance enhancement. Despite the valuable insights provided by these methods, further research is needed to explore the long-term effects of fiber reinforcement on FRCAC under varying dynamic loading conditions and to refine testing protocols for more accurate predictions of material behavior in real-world applications.

## 5. Mechanical Response Under Complex Loading Conditions

In practical settings, concrete structures frequently encounter multiple constraints and directions of complexity, underscoring the importance of investigating the mechanical characteristics of fiber-reinforced coral aggregate concrete under complex stress states for component analysis and structural design. Contemporary scholars have primarily focused on the performance of FRCAC under three complex loading conditions: combined compression–shear stresses, triaxial compression, and cyclic compression.

### 5.1. Combined Compression–Shear Stresses

**Figure 18:**
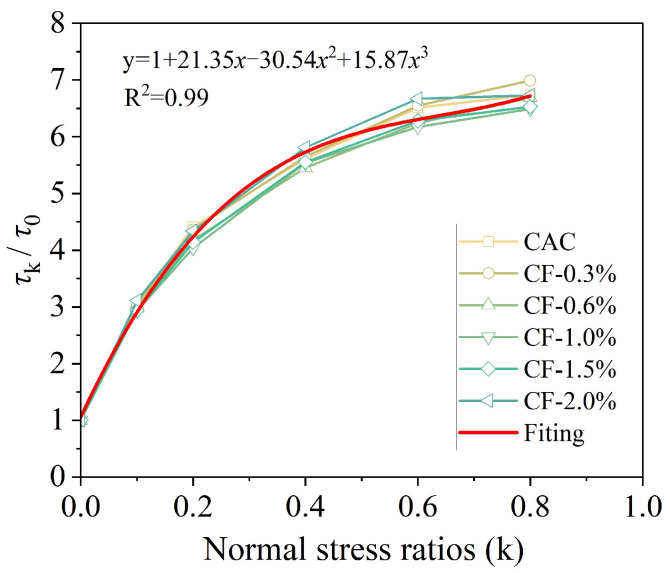
Axial compressive stress ratio and shear stress relationship [91].

Certain structural components, such as buttresses for highways, load-bearing piers, and deep beams, commonly experience combined compression–shear loading during operation [90]. Consequently, Liu et al. [91] focused on the compression–shear behavior of carbon-fiber-reinforced coral aggregate concrete (CFRCAC). The results of the experiments indicate that the compression–shear failure process of CFRCAC can be divided into three stages: elastic deformation, nonlinear deformation, and stress rapid drop. Further investigations have demonstrated that the normal stress ratio significantly affects the failure mode and shear strength of CFRCAC; an increase in the normal stress ratio results in a greater number of concrete fragments detaching along the primary shear crack surface owing to enhanced frictional interactions, thereby leading to an increase in shear strength. As shown in Figure 18, an increase in the normal stress ratio corresponds to an enhancement in the shear strength of the coral concrete, although the rate of increase in shear strength decreases. The shear strength is primarily influenced by the normal stress ratio and is not significantly affected by the fiber parameters. The incorporation of carbon fibers (CF) can mitigate the detachment of concrete fragments, although it has a minimal effect on the shear-damage surface. Furthermore, the addition of CF enhanced the shear strength, with an optimal proportion of approximately 1.5%. When the proportion of CF exceeded 1.5%, the reinforcing effect of CF diminished [92].

### 5.2. Uniaxial Cyclic Compression

Stress–strain behavior and damage evolution under cyclic loading significantly influence the load-bearing capacity, ductility, damage progression, and seismic assessment of concrete structures. Existing experimental studies have primarily been conducted under uniaxial cyclic compression conditions.

Results indicate that both polypropylene-fiber-reinforced coral aggregate concrete [93] and sisal-fiber-reinforced seawater-cured coral concrete [94] exhibit superior cyclic compression performance compared to conventional coral concrete. Although the degree of reinforcement varies with fiber type and dosage, fiber addition generally delays the deterioration of strength and stiffness, reduces the accumulation of plastic strain, and enhances deformation capacity and energy dissipation under cyclic loading conditions. This is due to the bridging and stitching effects of randomly distributed fibers within the concrete. To facilitate a clear comparison of the reported cyclic compression responses and key mechanical indicators, the principal experimental results from existing studies are summarized in Table 6.

### 5.3. Triaxial Compression

**Figure 19:**
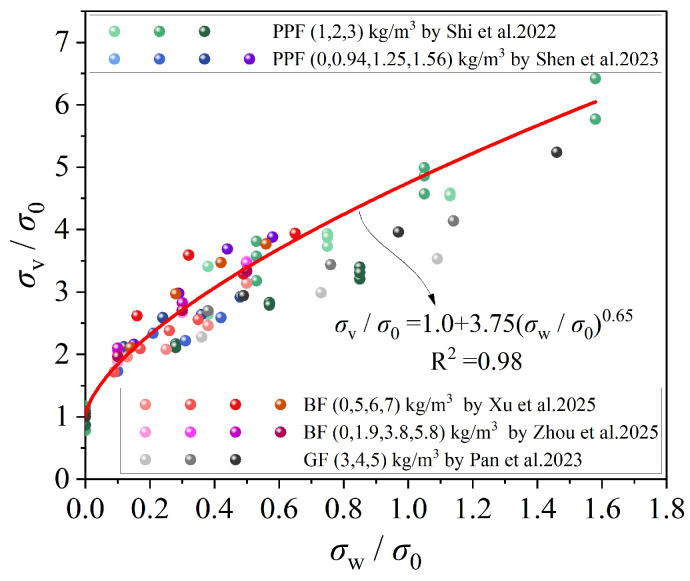
Peak stress and confining pressure relationship [95,96,97,98,99].

**Figure 20:**
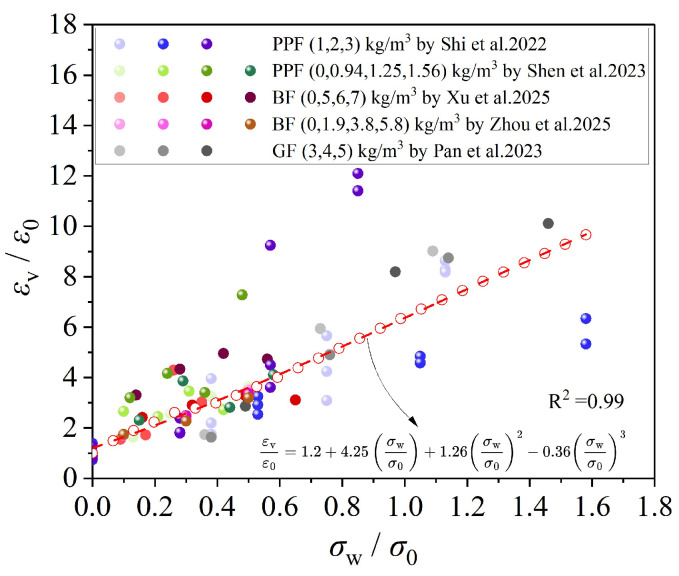
Peak strain and confining pressure relationship [95,96,97,98,99].

In practical engineering applications, concrete frequently operates under complex multi-axial stress conditions, which vary significantly from its mechanical behavior under uniaxial loading. Researchers have already performed conventional triaxial compression tests on FRCAC. In this study, previously reported experimental results on the triaxial compressive behavior of FRCAC were collected and analyzed based on the literature [95,96,97,98,99], as summarized in Figure 19 and Figure 20.

Figure 19 illustrates the relationship between axial compressive stress and confining pressure for FRCAC. The results indicate that the axial compressive strength is primarily governed by the confining pressure: higher confining pressure leads to greater axial load-carrying capacity, whereas the influence of fiber type and fiber content on strength is relatively limited. Among the fibers considered, GF exhibit a smaller contribution to the triaxial compressive strength of CAC compared to other fiber types. This behavior can be attributed to the restraining effect of confining pressure on the lateral expansion of CAC under compression, which significantly enhances both axial strength and compressive deformation capacity. Based on the statistical analysis of the collected data, an empirical relationship between the triaxial compressive strength of FRCAC and the confining pressure was established (Equation (1)), which can be used to predict the triaxial compressive strength of FRCAC.

(1) $$\frac{\sigma_{v}}{\sigma_{0}}=1.0+3.75{\left(\frac{\sigma_{w}}{\sigma_{0}}\right)}^{0.65}$$

Figure 20 presents the relationship between axial strain and confining pressure. The ultimate axial strain of FRCAC is strongly influenced by the confining pressure, and higher fiber contents generally result in improved deformation capacity under triaxial compression. This enhancement is mainly attributed to the ability of fibers to effectively suppress crack development induced by the second and third principal stresses (*σ*_2_ and *σ*_3_), thereby enabling the concrete to exhibit improved ductility. Based on the statistical results, a relationship between axial strain and confining pressure was also fitted (Equation (2)).

(2) $$\frac{\varepsilon_{v}}{\varepsilon_{0}}=1.2+4.25\left(\frac{\sigma_{w}}{\sigma_{0}}\right)+1.26{\left(\frac{\sigma_{w}}{\sigma_{0}}\right)}^{2}-0.36{\left(\frac{\sigma_{w}}{\sigma_{0}}\right)}^{3}$$

Overall, the available results demonstrate that triaxial confinement can significantly enhance both the load-carrying capacity and the axial strain of coral concrete. The incorporation of fibers provides an additional improvement in triaxial compressive performance, with a particularly pronounced contribution to deformation capacity. Therefore, fiber incorporation can be considered an effective modification strategy for improving the triaxial compressive behavior of CAC.

### 5.4. Summary of Complex Loading Conditions

Existing studies on FRCAC under complex loading conditions demonstrate that its mechanical response is strongly dependent on the applied stress state. Under combined compression–shear loading, fiber reinforcement primarily modifies damage evolution by restraining crack sliding and promoting more distributed cracking, while shear resistance remains predominantly governed by stress interaction effects. Under cyclic compression, fiber incorporation effectively mitigates stiffness degradation, strength deterioration, and damage accumulation through crack-bridging and energy dissipation mechanisms. In triaxial compression, confining pressure plays a dominant role in enhancing strength and altering failure modes, whereas fibers mainly contribute to improved deformation capacity and ductility.

Although the available experimental evidence remains limited, these studies collectively indicate that fiber reinforcement is most effective in regulating crack development and damage evolution rather than fundamentally altering strength-controlling mechanisms under complex stress states. Future research should focus on expanding the experimental database, clarifying fiber–stress coupling mechanisms, and developing unified constitutive models to support the reliable application of FRCAC in environments subjected to complex loading conditions.

## 6. Durability Performance

Durability is a critical factor governing the performance and service life of concrete structures, particularly in marine environments where exposure to aggressive agents, such as chlorides and sulfates, can accelerate material degradation. For FRCAC, ensuring adequate durability is essential for its application in coastal and offshore engineering. Among various durability-related issues, resistance to chloride ion penetration is widely recognized as a key indicator of long-term performance in seawater-exposed environments. Accordingly, this section reviews current studies on the durability of FRCAC, with a primary focus on chloride transport behavior and underlying influencing factors.

**Figure 21:**
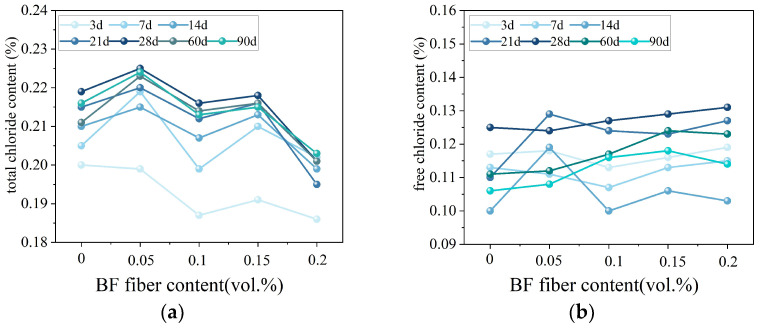
(**a**) The total chloride content and (**b**) free chloride content of all mixtures at different ages [25].

Existing studies indicate that fiber incorporation can improve the resistance of coral aggregate concrete (CAC) to chloride ion penetration under appropriate conditions. Fibers contribute to crack control and microstructural integrity by bridging microcracks and limiting crack connectivity, thereby reducing potential pathways for chloride ingress and ion exudation from porous coral aggregates. Wang et al. [41] reported that basalt fiber contents exceeding 0.05 vol% effectively inhibited chloride ion exudation from CAC. Similarly, Niu et al. [25] observed that while low basalt fiber dosages could initially accelerate chloride release, fiber contents in the range of 0.1–0.2 vol% significantly restrained chloride dissolution at later stages, and the chloride ion concentrations at different admixture ratios are shown in Figure 21. In contrast, Lu et al. [100] found that the addition of glass fibers had a negligible effect on chloride penetration resistance, suggesting that the durability response of FRCAC is strongly dependent on fiber type and fiber–matrix interaction mechanisms.

Overall, current research on the durability performance of FRCAC remains limited, and available findings are still dominated by short-term laboratory investigations. Although fibers have been shown to enhance crack resistance and may mitigate chloride transport under certain conditions, the long-term mechanisms governing durability improvement are not yet fully understood. In particular, the coupled effects of fiber characteristics, fiber–matrix interfacial behavior, and the inherently porous nature of coral aggregates require further clarification. Moreover, most existing studies focus on chloride ingress, whereas other durability-relevant processes under marine service conditions, such as sulfate attack, carbonation, abrasion, freeze–thaw behavior, and long-term fiber degradation, have received little systematic attention. Future studies should therefore emphasize long-term exposure tests under realistic marine environments and systematic comparisons of different fiber types and dosages to establish reliable durability design guidance for FRCAC.

## 7. Environmental Considerations and Sustainability of FRCAC

Although coral aggregates contribute to sustainability by repurposing natural resources, environmental concerns regarding coral mining and ecosystem disruption remain critical. According to Hossain et al. [101], the use of recycled aggregates, including coral waste, reduces energy consumption by 20–30% and carbon emissions by up to 25% compared to the use of virgin materials. These reductions are achieved by reusing materials that would otherwise lead to environmental degradation, in accordance with the Sustainable Development Goals. However, it is important to note that coral reefs, as ecosystems that support a large amount of marine biodiversity, are under serious threat from human activities, including coral mining. The State of the Caribbean Coral Reefs Report 1970–2012 by Jackson et al. [102] showed that coral cover in the Caribbean has declined by more than 50 per cent in recent decades and that direct coral mining is a significant contributor to this decline, with significant coral cover loss destroying marine habitats, affecting reef-dependent species, and triggering wider ecological consequences, such as reduced coastal storm protection [103].

It is essential to acknowledge the necessity of implementing appropriate management and protection measures when promoting the application of coral aggregate concrete. Such measures are critical to preventing the deliberate damage or overexploitation of coral reefs and ensuring a balance between ecological preservation and economic development. To achieve the sustainable development of construction projects located on remote islands and reefs while safeguarding fragile marine ecosystems, targeted management and protection strategies must be adopted.

First, the principles governing the utilization of coral aggregates should be clearly defined, as outlined in China’s ‘Technical Specification for the Application of Coral Aggregate Concrete’. From an ecological perspective, this specification emphasizes that coral aggregates should primarily be sourced from coral reef debris and that their development and use must follow rational and orderly practices to avoid adverse impacts on the marine environment. Second, further scientific research is required to improve the mechanical performance and durability of coral aggregate concrete, reduce its brittleness and susceptibility to corrosion, and enhance its suitability for engineering applications, thereby minimizing unnecessary resource consumption [104]. In parallel, the recycling of coral aggregate concrete should be encouraged, and the feasibility of using recycled coral aggregates warrants further investigation [105]. Finally, raising public awareness of environmental protection through education and outreach, and fostering societal support for coral reef conservation, are indispensable. This underscores the complex interaction between human activities and coral reef ecosystems, highlighting the importance of an integrated management approach that reconciles socio-economic development with ecological conservation [106].

In conclusion, the utilization of waste coral aggregates as construction materials for the development of remote islands is technically feasible and has the potential to offer environmental and economic benefits. When sourced in accordance with relevant regulations and environmental management guidelines, the reuse of coral reef debris can help avoid direct disturbance to intact reef ecosystems. Moreover, this approach may contribute to the conservation of land-based natural resources and reduce construction costs and environmental impacts associated with long-distance transportation. Nevertheless, the sustainable application of coral aggregate concrete relies on strict regulatory oversight, scientific management, and continuous monitoring to ensure that ecological risks are effectively controlled. Under these conditions, the rational use of waste coral aggregates represents a promising strategy for sustainable and resource-efficient construction in island and marine environments.

## 8. Conclusions and Future Research Directions

### 8.1. Conclusions

(1) The high porosity and water absorption of coral aggregates significantly weaken the mechanical strength and durability of coral aggregate concrete. Additionally, the ions in seawater used for mixing substantially alter cement hydration, accelerating early strength development but negatively impacting later-stage strength and long-term durability.
(2) Fiber incorporation significantly enhances the flexural and splitting tensile strengths of coral aggregate concrete, with reported improvement ranges of 0–67% and 0–58% (excluding basalt fibers), respectively, depending on fiber type, dosage, and matrix quality. In contrast, the effects on compressive strength and elastic modulus are generally limited, with variations of approximately −10% to 23% and −3% to 7%, respectively. Under dynamic and impact loading, fiber reinforcement further improves impact resistance and energy dissipation capacity. Accordingly, the reinforcing action of fibers in FRCAC is primarily associated with crack bridging and crack control mechanisms.
(3) Under complex loading conditions (combined compression–shear, triaxial compression, and cyclic loading), fibers contribute to reducing fragmentation and delaying damage evolution in coral concrete through crack-bridging action. Fiber reinforcement helps mitigate strength degradation under cyclic compression and can slightly modify failure modes under triaxial compression.
(4) Fiber incorporation can reduce crack connectivity and, to a certain extent, mitigate chloride ingress in coral aggregate concrete. However, under extreme marine environments and long-term service conditions, the durability mechanisms of FRCAC remain insufficiently understood and are not yet adequate to support reliable durability-oriented design.

### 8.2. Future Research Directions

(1) Future research should focus on conducting long-term exposure tests to systematically evaluate the service performance of FRCAC under realistic marine conditions, including wet–dry cycles, chloride and sulfate corrosion, and sustained loading. Continuous monitoring of performance changes in FRCAC components and structures within the marine environment is essential to validate laboratory findings and assess the actual behavior of FRCAC during service.
(2) Systematic investigations are required to optimize fiber geometry, dosage, and dispersion strategies in relation to matrix quality and coral aggregate characteristics. Emphasis should be placed on performance-based mix design approaches that balance mechanical enhancement, workability, and durability requirements, rather than relying solely on fiber type selection.
(3) The use of coral aggregates must be balanced with coral reef protection. Future FRCAC development should therefore prioritize efficient fiber utilization, recycled or bio-based fibers, and low-carbon binders to achieve both structural performance and environmental responsibility.

## Figures and Tables

**Table 1:** Physical properties of coral coarse aggregate.

Reference	Apparent Density (kg/m^3^)	Bulk Density (kg/m^3^)	Tube Compressive Strength (MPa)	l h Water Absorption (%)	24 h Water Absorption (%)	Porosity (%)
[25]	1724	909	2.27	-	15.0	47.3
[28]	1841	915	3.10	8.50	11.0	50.0
[31]	1966	938	3.07	8.26	-	52.3
[26]	1806	918	2.01	-	15.4	49.6

Note: A ‘-’ in the table indicates that the data were not available in the original literature.

**Table 2:** Physical properties of coral fine aggregate.

Reference	Apparent Density (kg/m^3^)	Bulk Density (kg/m^3^)	l h Water Absorption (%)	Mass Fraction %	
				Void	Clay
[32]	2350	1320	-	16.9	-
[33]	1538	1138	-	23.2	1.5
[34]	1638	1348	2.97	17.6	-
[27]	1659	1394	3.04	-	-

Note: A ‘-’ in the table indicates that the data were not available in the original literature.

**Table 3:** Properties of fibers used for reinforcing coral aggregate concrete.

Fiber	Category	Properties				Reference
		Density (g/cm^3^)	Tensile Strength (MPa)	Elastic Modulus (GPa)	Elongation at Break (%)	
Polypropylene fiber (PPF)	Organic fiber	0.91	694	4.24	15.4	[37]
Polyvinyl alcohol fiber (PVA)	Organic fiber	1.3	1600	42	-	[38]
Sisal fiber (SF)	Organic fiber	1.45	450~700	7~21	5~14	[39]
Carbon fiber (CF)	Inorganic fiber	1.82	4558	231	2.05	[40]
Basalt fiber (BF)	Inorganic fiber	2.56	2400	40	3.1	[41]
Glass fiber (GF)	Inorganic fiber	4.0	1700	-	-	[42]

Note: A ‘-’ in the table indicates that the data were not available in the original literature.

**Table 4:** Flexural behaviors of FRCAC.

Fiber Type	Fiber Content by Volume (%)	Coarse Aggregate	Fine Aggregate	Water/Binder Ratio	28 d Flexural Strength (MPa)	Reference
SF	0–0.67	Coral	river sand	0.40	2.72–4.04	[66]
PPF	0–0.45	Coral	Normal sand	0.40	2.70–4.50	[37]
CF	0–0.22	Coral	Normal sand	0.40	2.60–4.10	[76]
CF	0–0.7	Coral	coral sand	0.28–0.35	3.68–6.72	[40]
BF	0–0.2	Coral	coral sand	0.45	5.1–6.3	[75]

**Table 5:** Engineering-oriented summary of the reported effects of different fiber types on the basic mechanical properties of coral aggregate concrete.

Fiber Type	Typical Improvement in Compressive Strength	Typical Improvement in Splitting Tensile Strength	Typical Improvement in Flexural Strength	Effect on Elastic Modulus	Main Engineering Advantages	Main Limitations/Concerns
Polypropylene fiber (PPF)	−5%~18%	0%~38%	18%~67%	0%~4%	Effective crack control; significant improvement in flexural and tensile performance; good workability	Limited contribution to compressive strength and stiffness
Polyvinyl alcohol fiber (PVA)	−5%~22%	5%~18%	--	−3%~3%	Strong fiber–matrix bonding; enhanced ductility and post-cracking behavior	Higher cost; sensitive to fiber dispersion
Sisal fiber (SF)	0%~2%	7%~17%	19%~48%	1%~4%	Sustainable and renewable; effective crack-bridging in tension	Variability in fiber quality; durability under long-term exposure uncertain
Carbon fiber (CF)	1%~23%	1%~58%	1%~58%	−1~7%	High elastic modulus; effective stress redistribution; strong impact and tensile enhancement	High cost; dispersion and workability issues
Basalt fiber (BF)	−8%~15%	−12%~56%	0%~19%	2%~5%	Potential improvement in tensile performance and durability-related properties	Pronounced scatter; sensitive to fiber–matrix interaction and matrix quality
Glass fiber (GF)	−10%~9%	0%~29%	--	--	Uniform fiber distribution; moderate enhancement in tensile performance	Limited contribution to compressive strength; durability benefits remain unclear

Note: The enhancement ranges in the table were derived from experimental data distributions reported in Figure 5, Figure 9, Figure 10 and Figure 11. The values represent typical ranges rather than exact maxima or minima and are intended to reflect the general strengthening tendencies observed across multiple studies. The “--” in the table indicates that performance data for this item have not been collected.

**Table 6:** Uniaxial cyclic compressive behavior of FRCAC reported in the literature.

Fiber Type	Fiber Content by Volume (%)	Loading Regime	Key Observations	Reference
PPF	0.05–0.30 vol.%	Uniaxial cyclic compression	Peak stress increased by 10.45%, peak strain increased by 6.45%. Increasing fiber content reduces plastic strain accumulation and enhances the elastic stiffness ratio. Constitutive and damage models proposed.	[93]
SF	0.05–0.20 vol.%	Uniaxial cyclic compression	Peak stress increased by 2.34%, peak strain increased by 10.11%, maximum stiffness retention increased by 31.07%, and total energy dissipation increased by 51.36%. A stress–strain constitutive model and a damage evolution model were established.	[94]

## Data Availability

No new data were created or analyzed in this study. Data sharing is not applicable to this article.
